# PCNA accelerates the nucleotide incorporation rate by DNA polymerase δ

**DOI:** 10.1093/nar/gky1321

**Published:** 2019-01-03

**Authors:** Tanumoy Mondol, Joseph L Stodola, Roberto Galletto, Peter M Burgers

**Affiliations:** 1 Department of Biochemistry and Molecular Biophysics, Washington University School of Medicine, Saint Louis, MO, USA; 2 MilliporeSigma, St. Louis, MO, USA

## Abstract

DNA polymerase delta (Pol δ) is responsible for the elongation and maturation of Okazaki fragments in eukaryotic cells. Proliferating cell nuclear antigen (PCNA) recruits Pol δ to the DNA and serves as a processivity factor. Here, we show that PCNA also stimulates the catalytic rate of *Saccharomyces cerevisiae* Pol δ by >10-fold. We determined template/primer DNA binding affinities and stoichiometries by Pol δ in the absence of PCNA, using electrophoretic mobility shift assays, fluorescence intensity changes and fluorescence anisotropy binding titrations. We provide evidence that Pol δ forms higher ordered complexes upon binding to DNA. The Pol δ catalytic rates in the absence and presence of PCNA were determined at millisecond time resolution using quench flow kinetic measurements. The observed rate for single nucleotide incorporation by a preformed DNA-Pol δ complex in the absence of PCNA was 40 s^−1^. PCNA enhanced the nucleotide incorporation rate by >10 fold. Compared to wild-type, a growth-defective yeast PCNA mutant (DD41,42AA) showed substantially less stimulation of the Pol δ nucleotide incorporation rate, identifying the face of PCNA that is important for the acceleration of catalysis.

## INTRODUCTION

In eukaryotes, three DNA polymerases are involved in accurate and efficient DNA replication, DNA polymerase alpha (Pol α), DNA polymerase delta (Pol δ) and DNA polymerase epsilon (Pol ϵ) (1–3). Pol α which contains both polymerase and primase activities, synthesizes short RNA/DNA hybrid primers on the leading strand and initiates the synthesis of Okazaki fragments on the lagging strand. Pol ϵ is mainly responsible for the synthesis of leading strand (4), while Pol δ extends and matures Okazaki fragments on the lagging strand (3). *Saccharomyces cerevisiae* Pol δ is a heterotrimer consisting of three subunits: Pol3 (125 kDa), Pol31 (55 kDa) and Pol32 (40 kDa), present at a 1:1:1 stoichiometry (5).

The crystal structure of Pol3 catalytic subunit bound to a template/primer DNA has been determined previously (6). Small angle X-ray scattering shows that Pol δ containing a truncated form of Pol32 (amino acids 1-103) adopts an elongated conformation (7). However, detailed understanding of the overall structure of the Pol δ complex containing all three Pol3, Pol31 and Pol32 subunits in the presence of DNA remains incomplete. In part, these studies have been lacking because of the poor binding affinity of Pol δ for DNA. Its affinity for template/primer, double and single stranded DNA is much lower than that of yeast Pol ϵ (8,9). Previously, effort has been made to measure the binding affinity between Pol δ and DNA, but was unsuccessful due to the very weak binding of Pol δ to DNA (8).

Pol δ alone polymerizes DNA with low processivity (10–12), meaning that the number of nucleotides incorporated in a single polymerase-DNA binding event is very low. The proliferating cell nuclear antigen PCNA, a homotrimeric donut-shaped assembly that encircles DNA, increases the binding of Pol δ to the DNA (13), and this increases the processivity of Pol δ by at least a hundred fold (8,14). Similar observations have been made for Pol δ from other organisms (13,15,16).

Replication factor C (RFC) is the clamp loader that loads PCNA around double-stranded DNA at primer termini in an ATP-dependent reaction (17,18). The PCNA–Pol δ complex replicates DNA with high processivity. However, the activity of PCNA may not just be restricted to that of increased DNA binding and processivity. Two previous reports suggest that PCNA may also increase the actual rate of catalysis by the polymerase (9,19). In a recent study, we determined that at saturating dNTP concentrations, the rate of incorporating a single nucleotide by the PCNA–Pol δ complex was too fast to measure accurately by the quench-flow technique employed (>300 s^−1^) (9). However, without PCNA, that same incorporation event was measured at 9 s^−1^ (9). Another pre-steady state kinetic study of Pol δ alone indicated a rate of incorporation of a single nucleotide at ∼1 s^−1^ at saturating dNTPs (19). Both studies suggest that, since replication fork movement inside yeast proceeds at about 50 nucleotides per second (20), PCNA would have to increase the catalytic rate of nucleotide incorporation by Pol δ substantially.

Given the large discrepancy between different reports of the catalytic rates of Pol δ alone, we decided to re-investigate this issue under conditions at which burst-phase kinetics could be applied appropriately. In order to ensure that our kinetic analysis would actually measure the rate of catalysis by a stable, preformed DNA-Pol δ complex, we first examined the stoichiometry of Pol δ to template/primer DNA using electrophoretic mobility shift assays, fluorescence anisotropy and fluorescence quenching methods. Our studies provide evidence that the stoichiometry of Pol δ binding to template/primer DNA is two. Next, we measured the nucleotide incorporation rate by Pol δ under conditions of burst-phase kinetics. At saturating dNTPs this rate is 40 s^−1^, and in the presence of PCNA, this rate increases to >350 s^−1^, indicating strongly that PCNA increases the nucleotide incorporation rate by an order of magnitude. Finally, we have identified a PCNA mutant, pcna-6 (DD41,42AA), which increased the rate of nucleotide incorporation substantially less than wild-type PCNA.

## MATERIALS AND METHODS

### Proteins

DNA polymerase δ (Pol δ-DV) and a variant version of Pol δ (mini-Pol δ-DV) purified from a yeast overexpression system (21). Replication protein A (RPA) (22), wild-type proliferating cell nuclear antigen (PCNA) (23), pcna-6 mutant (24) and replication factor C (RFC) (25) were overexpressed and purified from *Escherichia coli*. The mini-Pol δ-DV enzyme was used in most reactions, unless otherwise noted. The exonuclease-deficient mutant (D520V) prevents degradation of oligonucleotide substrates (26). AMP-CPP was purchased from Sigma-Aldrich.

### DNA substrates

Purified oligonucleotides were purchased from Integrated DNA Technologies (IDT). Template DNA strand for primer extension reaction of more than one nucleotide was labelled with biotin in both ends. Primers used for all the primer extension assay were labelled with a 5′-Cy3 fluorophore. Oligonucleotides were hybridized by heating to 85°C for 5 min in 100 mM NaCl and then cooling down slowly to room temperature. The concentration of streptavidin added was twice that of the biotinylated template/primer substrates. The DNA substrate used in Figures 3 and 4 contains 3′ and 5′-biotin–streptavidin bumpers in order to constrain PCNA onto the DNA.

**Figure 1.**
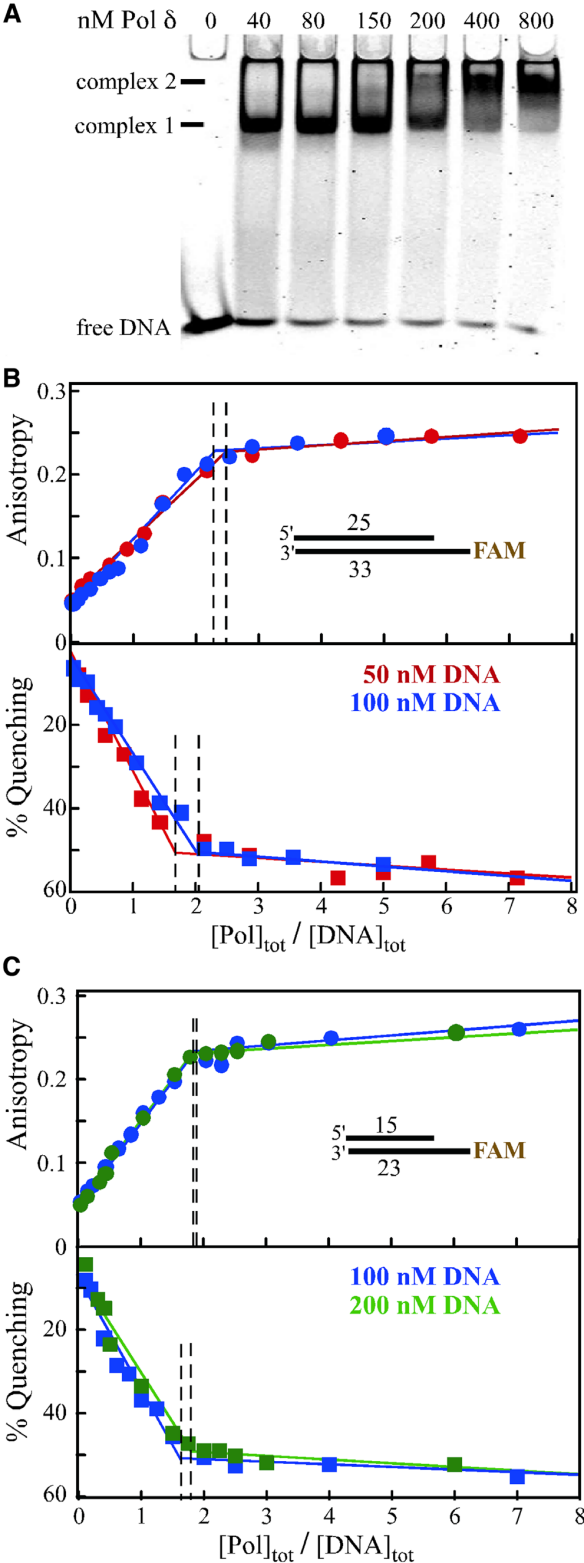
DNA binding studies of Pol δ. (**A**) Electrophoretic mobility shift assay. 25 nM 33/25 template/primer DNA was incubated with increasing concentrations of mini-Pol δ-DV at 100 mM NaCl. Complexes were resolved on a 5% native polyacrylamide gel (see Materials and Methods). Complex 1 and 2 appear successively at increasing polymerase concentrations. (**B, C**) Fluorescence anisotropy and fluorescence quenching studies. The indicated template/primers (33/25 in (B) and 23/15 in (C)) were mixed with increasing concentrations of mini-Pol δ-DV at 40 mM NaCl. In (B) 50 nM DNA in red, 100 nM DNA in blue; in (C) 100 nM DNA in blue, 200 nM DNA in green. The data points are fitted to a two-segment fit analysis (using Origin software). Vertical dotted lines were drawn from the intersection points.

Template/primer (33/25) DNA sequence used for EMSA and fluorescence anisotropy and fluorescence quenching studies in Figure 1 is: 25-mer primer: 5′-CGTAACGCTACCGCAGCTGCAGCTG-3′, 33-mer template: 5′-FAM-TTTTTTTTCAGCTGCAGCTGCGGTAGCGTTACG-3′.

**Figure 2.**
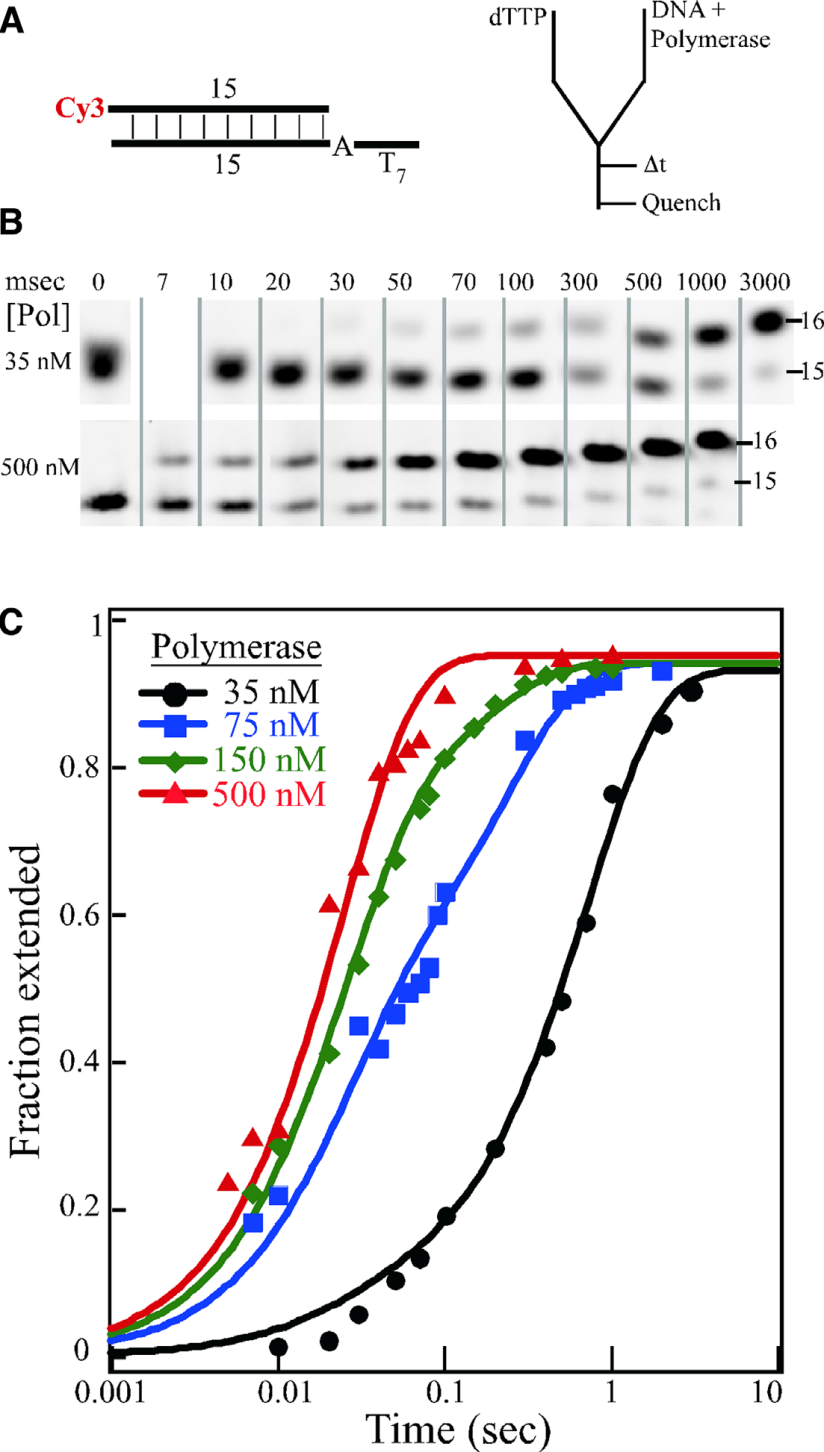
Single nucleotide extension studies. (**A**) DNA substrate and rapid quench experimental setup. (**B**) Selected time points of single nucleotide extension by 35 and 500 nM mini-Pol δ-DV, pre-incubated with 50 nM 23/15 template/primer DNA. Reactions were initiated with 250 μM final dTTP. The full gels are shown in Supplementary Figures S4A and S4B. (**C**) Quantification of the data for 35 nM (black), 75 nM (blue), 150 nM (green) and 500 nM (red) mini-Pol δ-DV. The fractional extension of the 15-mer product is plotted against time on a logarithmic scale.

Template-primer (23/15) DNA sequence used for polymerase single nucleotide extension assay in Figure 2 is: 15-mer primer: 5′-Cy3-GCTGCTCGGTCTCGC-3′, 23-mer template: 5′-TTTTTTTAGCGAGACCGAGCAGC-3′. For fluorescence anisotropy and fluorescence quenching studies in Figure 1, FAM as sole fluorophore was at the 5′-template position.

**Figure 3.**
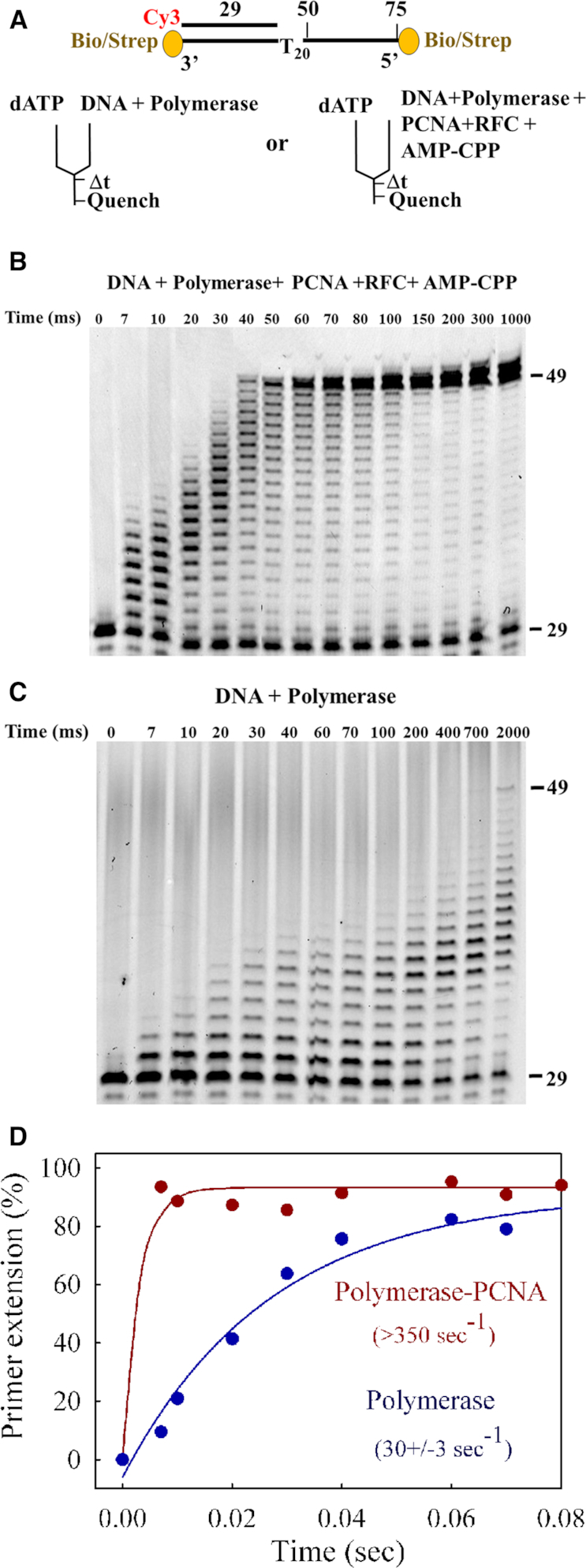
20-nucleotide replication assays. (**A**) DNA substrate and rapid quench experimental setup. The 75-mer template contains double biotin-streptavidin blocks to prevent PCNA from sliding off the DNA. (**B**) Time course of primer extension by 300 nM mini-Pol δ-DV pre-incubated with 50 nM DNA in presence of 150 nM PCNA, 75 nM RFC and 100 μM AMP-CPP. (**C**) Time course in the absence of PCNA, RFC and AMP-CPP. Both reactions were initiated with 250 μM final dATP. The complete data sets are shown in Supplementary Figures S4C and S4D. (**D**) Quantification of the data. Primer extension is plotted against time in the absence (blue) and in the presence (red) of PCNA, using single exponential fittings.

**Figure 4.**
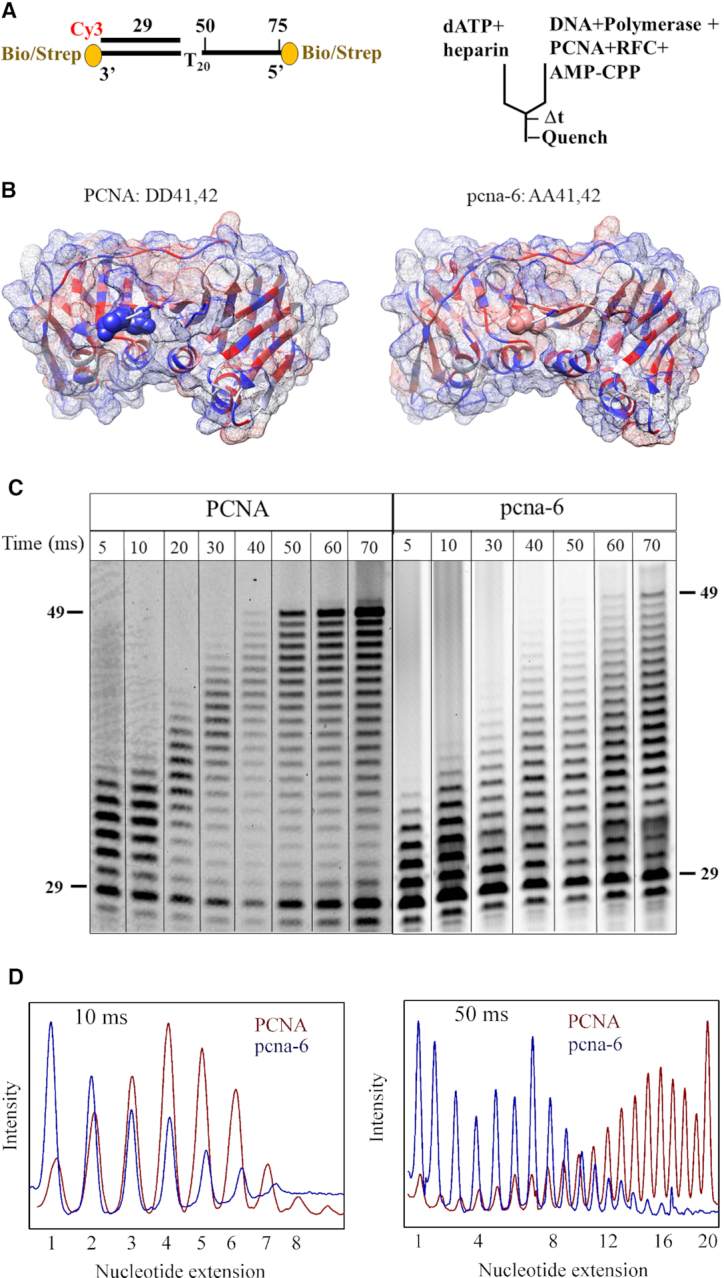
Replication defects of pcna-6 (DD41,42AA). (**A**) DNA substrate and rapid quench experimental setup (as in Figure 3, except for the addition of a heparin trap). (**B**) Crystal structure of wild-type PCNA (1PLR) with D41 and D42 shown as CPK in blue and pcna-6 (5V7K) with A41 and A42 shown in pink. Hydrophilic surface in blue and hydrophobic surface in red. (**C**) Time courses of primer extension by mini-Pol δ-DV (300 nM) pre-incubated with 50 nM DNA in the presence of 75 nM RFC and 100 μM AMP-CPP, and either 150 nM PCNA (left), or 150 nM pcna-6 (right). Reactions were initiated with 250 μM final dATP and 17 μg/ml final heparin sulphate. Complete data are in Supplementary Figures S4E and S4F. (**D**) Comparison of product distribution with PCNA (red) and pcna-6 (blue) at 10 and 50 milliseconds. Normalized band intensities are shown with the highest intensity for each distribution set to 1.

Template/primer (75/29) DNA sequence used for polymerase primer extension assay in Figure 3 and in Figure 4, is: 29-mer primer: 5′-Cy3-TCA GCG CGA GCA TGA CAT TGA AGG TAA CC-3′, 75-mer template: 5′-BiotinTEG-TTC CTT CAA CCA GCT TAC CTT CTT CCT TTT TTT TTT TTT TTT TTT TGG TTA CCT TCA ATG TCA TGC TCG CGC TGA-BiotinTEG-3′.

**Figure 5.**
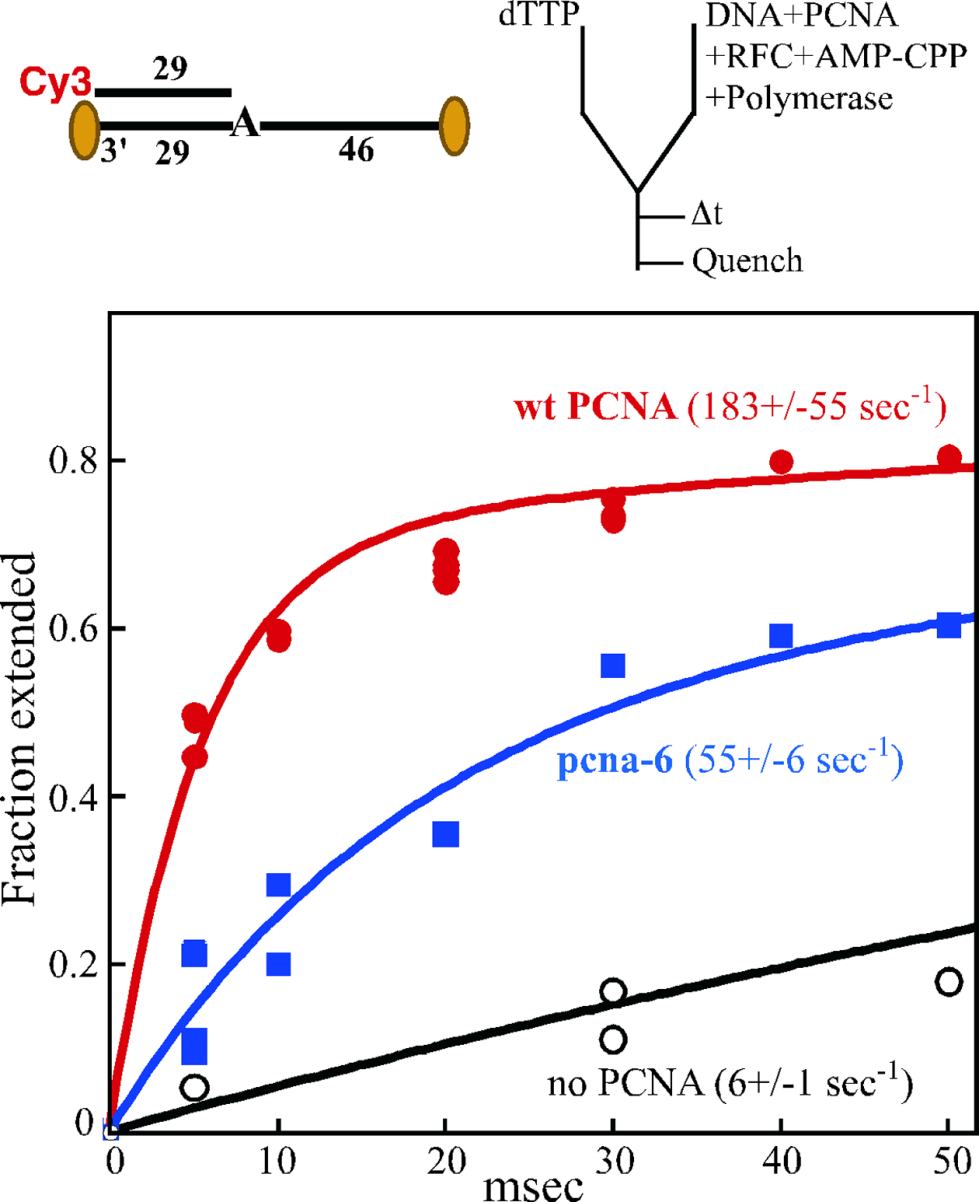
Single-nucleotide extension defect of pcna-6 (DD41,42AA) at 12°C. Top, substrate and experimental setup are identical to described in Figure 3, except that the 76/29 substrate has a single template adenine residue, and we used full-length Pol δ-DV. Upon addition of dTTP to the preformed complex, extension is by one single nucleotide. Assays were performed at 12°C. Bottom, primer extension is plotted against time in the absence of PCNA (black), with wild-type PCNA (red) or with pcna-6 (blue). The data were modeled to the sum of two exponentials, and *k*_fast_ is shown (see Materials and Methods). For the assay without PCNA, the assay was taken out to 1 s, at which time 84% extension had occurred. These data were included in the modelling, but only the portion up to 50 ms is shown.

Template/primer (76/29) DNA sequence used for polymerase primer extension assay in Figure 5, is: 29-mer primer: 5′-Cy3-TCA GCG CGA GCA TGA CAT TGA AGG TAA CC-3′, 75-mer template: 5′-BiotinTEG-TTC CTT CAA CCA GCT TAC CTT CTT CCT TTT TTT TTT TTT TTT TTT TAGG TTA CCT TCA ATG TCA TGC TCG CGC TGA-BiotinTEG-3′

### Replication reactions

All the primer extension assays were performed using a rapid chemical quench flow apparatus (Kintek RQF-3) maintained at 12 or 30°C with a circulating water bath.

Single nucleotide incorporation assays of mini-Pol δ-DV were performed in a buffer containing 40 mM Tris–HCl, pH 7.8, 1 mM DTT, 200 μg/ml bovine serum albumin, 8 mM MgCl_2_ and 40 mM NaCl. Both syringes in the quench-flow setup contained this buffer. In one syringe, 100 nM DNA template was pre-incubated with various concentration of mini-Pol δ-DV (70 nM-1000 nM). The other syringe contained 500 μM dTTP. The reactions were initiated by mixing equal volumes and the reactions were quenched at different time intervals by adding 0.2 M EDTA. The DNA products were resolved on 15% denaturing polyacrylamide gels. Gels containing Cy3-labeled DNAs were visualized by detection of Cy3 fluorescence with a Typhoon-Trio (GE Healthcare). All quantifications were carried out with ImageQuant software (GE Healthcare). Typhoon gel images were subjected to a linear transformation for presentation in figures using ImageJ.

Primer extension assays in the presence of PCNA were performed in the same reaction buffer, except for 100 mM NaCl. One syringe contained 100 nM template/primer DNA, 600 nM mini-Pol δ-DV, 300 nM PCNA (or pcna-6), 150 nM RFC, 200 μM AMP-CPP. The second syringe contained 500 μM dATP in the same buffer and the reaction was initiated by mixing equal volumes. When PCNA was omitted, RFC and AMP-CPP were also omitted. In some assays, heparin sulphate (34 μg/ml) was added to the dATP-containing syringe as a trap for dissociated polymerase molecules. Separation of products and detection was as described above. Changes from this protocol are indicated in the legends to figures.

To determine the polymerase rate constants in Figure 2, the fraction of the product formed was plotted as a function of time and fitted to the following equation:

\begin{equation*}\ \left[ {Product} \right] = \ A\left( {1 - {e^{ - {k_{fast}}t}}} \right) + \left( {1 - A} \right)\left( {1 - {e^{ - {k_{slow}}t}}} \right)\end{equation*}

where *k*_fast_ is the fast phase with amplitude *A* and *k*_slow_ represents the slow phase with amplitude (1 – *A*). When substrate DNA was saturated with polymerase at 500 nM, the rate was fitted to a single exponential function.

### Electrophoretic mobility shift assay (EMSA)

The EMSA of mini-Pol δ-DV with FAM labelled template/primer 33/25 DNA was performed on 5% native polyacrylamide gel in 1× Tris/borate/EDTA (TBE) running buffer pre-chilled at 4°C. Gels were scanned using Typhoon-Trio (GE Healthcare).

### Fluorescence intensity and anisotropy titrations

All fluorescence titrations were performed with an L-format PC1 spectrofluorimeter (ISS, Champaign, IL) equipped with Glenn-Thompson polarizers. Measurements of the anisotropy and total fluorescence intensity of FAM-labelled DNA were recorded using excitation and emission wavelengths of 480 and 520 nm respectively. Anisotropy was measured using the equation

\begin{equation*}r\ = \frac{{{I_{{\rm VV}}} - G{I_{{\rm VH}}}}}{{{I_{{\rm VV}}} + 2G{I_{{\rm VH}}}}}\ \end{equation*}

where *I*_TOT_ = *I*_VV_ + 2*GI*_VH_; *I*_VV_ and *I*_VH_ are the fluorescence intensities of vertically and horizontally polarized emission when the fluorophore is excited with vertically polarized light and *G* is the *G* factor (27). The change in *I*_TOT_ relative to the value of DNA only is reported. Titrations were performed with quartz cuvette having path length of 1 cm.

## RESULTS AND DISCUSSION

### Replication efficiency of a Pol δ variant

In order to focus our study on the polymerization activity of Pol δ without complications due to its proofreading activity, we used the exonuclease-deficient Pol δ-DV (D520V) heterotrimer, overproduced in *S. cerevisiae* (26). Preliminary DNA binding studies indicated that we would need to use μM concentrations of Pol δ to achieve saturation binding, which were difficult to achieve experimentally because of a tendency of Pol δ-DV to aggregate at such high concentrations. Therefore, we made a variant version of Pol δ, called mini-Pol δ-DV, which could be purified more easily to higher concentrations, and without detectable aggregation. Mini-Pol δ-DV differs from wild-type in the following respects: first, an internal truncation derivative of Pol32, i.e. Pol32-11 (amino acids 1–142 + 310–350, lacking amino acids 143–309) was used instead of full-length. Pol32 has an extremely elongated shape (5,7). Study of *S. pombe* Pol δ showed that the elongated structure of its third subunit Cdc27 was mainly caused by the central portion of this subunit and a deletion variant of Cdc27 missing the central 110 amino acids was much more globular in structure (16). The biochemical properties of Pol δ containing Pol32-11 are comparable to that of wild-type, and the mutant is phenotypically indistinguishable from wild-type Pol32, with regard to growth, damage sensitivity and mutagenesis (28). Second, we used a Pol3 N-terminal truncation of 81 amino acids. The N-terminal ∼100 amino acids of the catalytic subunit Pol3 is unstructured and not conserved. Crystal structure determination of the Pol3-DNA-dNTP complex was carried out with an N-terminal 68 amino acid truncation, but the region up to position 95 remained unresolved and likely unstructured (6). No observable growth and DNA damage response phenotypes were associated with this truncation (data not shown). These two changes allowed us to isolate a smaller, more globular version of Pol δ. We designate this variant as mini-Pol δ-DV.

We obtained consistently a few fold higher yield of mini-Pol δ-DV compared to full-length Pol δ-DV after overexpression. Mini-Pol δ-DV contains the three subunits in approximately equimolar ratio (Supplementary Figure S1A), with a similar [4Fe–4S] cluster content as wild-type (29), as evident from its characteristic UV absorption at 410 nm (data not shown). We compared the replication activities of mini-Pol δ-DV with Pol δ-DV using an *in vitro* replication assay. PCNA was loaded by RFC and ATP onto primed circular single-stranded DNA (M13mp18 DNA) coated with the single-stranded DNA binding protein replication protein A (RPA). The kinetics of processive DNA synthesis by the two forms were compared by resolving replication products using denaturing agarose gel electrophoresis (Supplementary Figure S1B). Both enzymes showed comparable activities. All our DNA binding studies were carried out with mini-Pol δ-DV. Key binding experiments were also carried out with full-length Pol δ-DV, and these are presented in supplementary figures.

### DNA binding properties and stoichiometry of Pol δ

Initially, we investigated the binding of Pol δ to carboxy-fluorescein (FAM) labelled template/primer DNA (33/25) using electrophoretic mobility shift assay (EMSA). DNA (25 nM) was incubated with increasing concentrations of polymerase and the DNA-protein complexes were resolved on a 5% native polyacrylamide gel. Two DNA-polymerase complexes were observed, depending on the molar excess of Pol δ (Figure 1A), suggesting the binding of multiple Pol δ molecules at higher concentrations.

However, since EMSA may not always accurately reflect the true affinity of a protein for DNA (30), we turned to a methodology where we could directly measure this affinity under the same solution conditions that would be used in our kinetic assays of polymerase activity. We performed equilibrium binding studies of Pol δ to the 33/25 template/primer, monitoring the change of fluorescence anisotropy and total fluorescence of FAM-labelled substrates as a function of Pol δ concentration. The binding of Pol δ to DNA is accompanied by a large change in fluorescence anisotropy. Supplementary Figure S2A shows the change in fluorescence anisotropy of the FAM labelled template/primer DNA (33/25) as a function of polymerase concentration at 40 mM and 100 mM NaCl. The anisotropy of the DNA gradually increases with increasing polymerase concentration as the rotation of the fluorophore in the polymerase-DNA complex decreases compared to free DNA. At 100 mM NaCl, saturation binding could not be achieved, even at the highest enzyme concentration used (1.4 μM). Therefore, these conditions are inappropriate for measuring polymerization activity of a preformed DNA–Pol δ complex, and we repeated the binding experiments at 40 mM NaCl. Now, saturation binding was reached at ∼250 nM enzyme, and we used these exact same conditions in our replication studies.

At saturating protein concentrations, the inflection points in the increase in anisotropy and the accompanying fluorescence quenching indicate that approximately two molecules of Pol δ bind to the 33/25-mer substrate, independent of the DNA concentration used (Figure 1B). When the double-stranded DNA section was reduced from 25 base pairs to 15 base pair, very similar results were obtained (Figure 1C). Again, the data are most consistent with the binding of two molecules of Pol δ to the 23/15 substrate. In addition, the same 2:1 stoichiometry was also observed with full-length Pol δ, indicating that binding of the second molecule is not an artifact introduced by the deletions in the mini-Pol δ variant (Supplementary Figure S2B, C).

Based on structural studies, these results suggest that the second polymerase may not bind to the template/primer region itself. The crystal structure of the ternary complex of the Pol3 catalytic domain with template/primer DNA and an incoming base-paired dNTP reveals that the Pol3 domain occludes about 8 nucleotides of dsDNA and an estimated five nucleotides of ssDNA (6). Based on small angle X-ray scattering analysis of the complex, the length of DNA occluded by the three-subunit Pol δ is proposed to be even larger because of the presence of a DNA binding domain in Pol31 (7). Control experiments showed that Pol δ also bound both single- and double-stranded DNAs, with binding to blunt-end double-stranded DNA being the weakest (Supplementary Figure S3). Therefore, a possible binding of the second Pol δ molecule to the DNA blunt end cannot be excluded. Alternatively, while Pol δ itself is a monomeric assembly in the absence of DNA (5), the possibility cannot be excluded that DNA-induced dimerization of Pol δ occurs, with the second molecule not being in direct contact with the DNA. This phenomenon has been observed for other DNA polymerases. For instance, oligomerization of an archaeal DNA polymerase is associated with an increased processivity of the enzyme (31,32).

At the 40 mM NaCl concentration used in our binding studies, the interactions are tight, preventing us to reliably estimate the equilibrium binding constants for binding of the first and second polymerase molecule and thus, a detailed model for the mode of binding of Pol δ to these substrates remains to be determined. Notwithstanding, both EMSA and equilibrium binding experiments indicate that at 40 mM NaCl, two molecules of Pol δ likely bind to template/primer DNA.

### Pre-steady state kinetic studies of nucleotide incorporation rate by Pol δ in absence of PCNA

Pol α, δ and ϵ are members of the B-family DNA polymerase, to which also belong bacteriophage T4 and Rb69 DNA Polymerase (33,34). A minimal kinetic scheme for dNTP incorporation by a B-family DNA polymerase involves binding of the enzyme to DNA (k1), binding of the complementary dNTP (k2), a conformational change from an open to a closed conformation (k3), and the chemical step (k4). All steps are reversible.

\begin{equation*}\begin{array}{@{}*{1}{l}@{}} {{\rm{DN}}{{\rm{A}}_{\rm{n}}} + {\rm{Pol\ }}\begin{array}{@{}*{1}{c}@{}} {{{\rm{k}}_1}}\\ \rightleftharpoons \\ {{{\rm{k}}_{ - 1}}} \end{array}{\rm{DN}}{{\rm{A}}_{\rm{n}}}\cdot{\rm{Pol}}\begin{array}{@{}*{1}{c}@{}} {{{\rm{k}}_2}}\\ \rightleftharpoons \\ {{{\rm{k}}_{ - 2}}} \end{array}{\rm{\ DN}}{{\rm{A}}_{\rm{n}}} \cdot {\rm{Pol}} \cdot {\rm{dNTP}}}\\ {{\rm{\ }}\begin{array}{@{}*{1}{c}@{}} {{{\rm{k}}_3}}\\ \rightleftharpoons \\ {{{\rm{k}}_{ - 3}}} \end{array}{\rm{DN}}{{\rm{A}}_{\rm{n}}}\cdot{\rm{Po}}{{\rm{l}}^{\rm{*}}}\cdot{\rm{dNTP\ }}\begin{array}{@{}*{1}{c}@{}} {{{\rm{k}}_4}}\\ \rightleftharpoons \\ {{{\rm{k}}_{ - 4}}} \end{array}{\rm{\ DN}}{{\rm{A}}_{{\rm{n}} + 1}}\cdot{\rm{Po}}{{\rm{l}}^{\rm{*}}}\cdot{\rm{PPi}}} \end{array}\end{equation*}

For Pol δ, this scheme is more complex because of the binding of two enzyme molecules to the DNA, each likely with a different *K*_D_ (Figure 1A). However, at saturating enzyme concentrations, this complexity disappears. A kinetic analysis of T4 DNA polymerase showed that when the enzyme was pre-bound to DNA, the rate of dNTP incorporation could be determined using burst-phase kinetics, and it was 400 s^−1^ (35). Similar polymerization rates were measured for yeast Pol ϵ and for Rb69 DNA polymerase (Table 1). Given these very high catalytic rates, the low rates obtained in the previous kinetic analyses of Pol δ have been very perplexing. However, single-nucleotide incorporation rates as determined by burst-phase kinetics are only interpretable if indeed the DNA were saturated with Pol δ upon addition of the complementary dNTP.

**Table 1.** Comparison of nucleotide incorporation rate (*K*_pol_) of different B-family replicative DNA polymerases. Rates were determined using burst-phase kinetics at 25°C (Rb69, T4), or 30°C (Pol δ, Pol ϵ)

Polymerase (Ref)	*K*_pol_ (sec^−1^)
Pol δ (this study)	40±3
Pol δ + PCNA ((9), this study)	>350
pol ϵ (42)	319±5
T4 (35)	400
Rb69 (53,54)	320±25

In one previous study, yeast Pol δ (60 nM) was pre-bound to 150 nM DNA in 150 mM sodium acetate buffer (19). Upon addition of dNTP, the fast phase of 1 s^−1^ was taken to represent burst-phase kinetics. However, given our DNA binding data, it is unlikely that there was substantial enzyme-DNA complex formation at that salt concentration. Similarly, in a recent study from our own group (9), we determined by EMSA that at 100 mM NaCl, 40 nM Pol δ-DV completely shifted 10 nM DNA into a complex, suggesting that burst-phase kinetics could be applied under those conditions. The observed catalytic rate was 9 s^−1^. Again, our fluorescence anisotropy data (Supplementary Figure S2A) suggest that the EMSA methodology incorrectly reported on enzyme–DNA binding affinity, and binding was not saturating under those conditions.

We have reinvestigated the rate of incorporation of a single nucleotide by Pol δ, using the rapid quench flow apparatus. In order to ensure full occupancy of DNA with polymerase, we have performed the pre-steady state kinetic measurements at 40 mM NaCl, because at this salt concentration saturation binding by polymerase can be achieved. We pre-incubated 100 nM 23/15 template/primer DNA with increasing polymerase concentrations from 70–1000 nM and started the reaction by mixing with an equal volume of 500 μM dTTP (Figure 2A). Therefore, final concentrations in the assay were 50 nM DNA, 35–500 nM enzyme and 250 μM dTTP, and these numbers are used in the figures and in the discussion below. We monitored extension of the 15-mer primer by one single nucleotide (Figure 2B). Since our binding studies show that at 500 nM Pol δ, all DNA is in the polymerase-bound form, the condition of burst kinetics applies. Therefore, the observed rate is determined by k3 and k4 (dNTP binding (k2) is not rate limiting at the saturating concentration of 250 μM). We fitted the data points to a single exponential, which yielded a burst rate of 40 ± 3 s^−1^ (Figure 2C).

The data points for the 35, 75 and 150 nM concentrations were fitted to a sum of two exponentials (Figure 2C). The fast phase was taken from the experiment with 500 nM Pol δ and represents the activity of the pre-formed Pol•DNA complex with a rate of 40 s^−1^, whereas the slow phase includes contributions of polymerase binding to template/primer (k1) followed by catalysis (k3 and k4). This analysis allowed us to determine the fraction of DNA bound up in a complex that results in a fast phase, and the fraction of DNA that represents the slow phase (Supplementary Table 1). These data indicate about half-saturated binding at 75 nM Pol δ, which agrees well with the binding isotherms in Figure 1. Importantly, the overall kinetics for extension were not substantially higher at 500 nM than at 150 nM Pol δ, indicating that saturation had been reached (Figure 2C). In our analysis, we have not taken into consideration the presence and activity of DNA with only one polymerase bound. Whereas it is clear that at 500 nM Pol δ, the complex has a stoichiometry of Pol_2_-DNA_1_, at lower concentrations there could be mixture of Pol_2_-DNA_1_, Pol_1_-DNA_1_, and free DNA. Currently, the methodology does not allow us to distinguish between these possibilities. Therefore, for this study, we have primarily limited ourselves to experiments in which the DNA is saturated with Pol δ in a 1:2 complex.

Remarkably, the *k*_fast_ rate constant for Pol δ is much lower than those observed for other replicative polymerases in this family (Table 1). If one considers that the observed rate of 40 s^−1^ has been determined at saturating dNTP, and that the intracellular dNTP concentrations of 12–30 μM are far below *K*_m_ values (19,36), it becomes evident that polymerization by Pol δ alone is too slow to sustain a fork rate of ∼50 nucleotides per second (20). And since recently we measured replication rates of the PCNA–Pol δ complex at >300 nucleotides per second (9), it is evident that PCNA must increase the catalytic rate of Pol δ.

### PCNA stimulates the catalytic rate of Pol δ

We have previously set up a quench-flow system in order to measure DNA replication in the presence of PCNA (9). Because the rate of single nucleotide addition was too rapid to be accurately measurable with our apparatus, we used a T_20_ template stretch, which lacks secondary structure. Upon addition of dATP, replication of 20 nucleotides occurred monotonously (Figure 3A). Briefly, the experimental set-up uses a template/primer (75/29) DNA with biotin-streptavidin blocks at both ends in order to constrain PCNA onto the DNA. Furthermore, PCNA is loaded by RFC and the ATP analog AMP-CPP, which has a methylene group bridging the α- and β-phosphates. This analog supports efficient PCNA loading by RFC, but does not serve as a substrate for incorporation by Pol δ. When we pre-incubated DNA with RFC, PCNA and AMP-CPP to load PCNA, and with Pol δ to pre-form the ternary PCNA–DNA–Pol δ complex, replication occurred rapidly upon addition of dATP (Figure 3B). The experiments were performed at 100 mM NaCl. The fastest moving complexes reached the end of the T_20_ template after only 40 millisecond, a rate of 500 nucleotides per second. In sharp contrast, when PCNA and RFC were omitted from the pre-incubation, extension was slow, and complete synthesis of the T_20_ template was only accomplished after a prolonged incubation for 2 s (Figure 3C). This large decrease in the observed rate is due to a combination of slow polymerization without PCNA and low processivity, requiring multiple rebinding events of the polymerase in order to replicate the full T_20_ template. We quantified the rate of disappearance of the primer, a variable that is not affected by polymerase processivity. These data were fitted to a single exponential equation yielding a minimal catalytic rate of 350 s^−1^ by the PCNA–Pol δ complex (Figure 3D), in excellent agreement with earlier studies (9). Without PCNA, saturation of the template/primer could not even be achieved at the large excess of Pol δ used in this experiment (Supplementary Figure S2A). Therefore, the calculated rate of 30 sec^−1^ must be a lower estimate. Nevertheless, it compares well with the rate of 40 s^−1^ obtained at 40 mM NaCl (Table 1).

The PCNA-mediated acceleration in catalysis is unique for Pol δ. It was not observed in the best studied B-family polymerase and circular clamp system, that from bacteriophage T4 (37–40). The PCNA-like T4-gp45 clamp stimulates the processivity of T4 DNA polymerase. However, the intrinsic rate of T4 DNA polymerase is already high (Table 1), and it is not significantly accelerated by the gp45 clamp (37–40). Similarly, the intrinsic catalytic rates of other replicative B-family polymerases are also very high (Table 1), making it unlikely that the clamp would further stimulate catalysis.

In the minimal kinetic scheme, two steps are likely candidates to be rate-limiting for catalysis by a DNA•Pol complex: (i) that of the conformational change of the ternary DNA•Pol•dNTP complex from an open to a closed state in which the thumb domain and the fingers domain are brought in close proximity (k3); or (ii) that of the actual catalytic step (k4). For the well-studied A-family replicative DNA polymerase from bacteriophage T7, the conformational change k3 was shown to be the rate-limiting step (41). In the T4 system, the data did not allow a conclusion to be made regarding the rate-limiting, k3 or k4 (35). However, in a recent kinetic study of yeast Pol ϵ, it was shown that the chemical step k4 is not the rate-limiting step, suggesting that the conformational change k3 most likely is (42).

### A PCNA mutant is partially defective in accelerating the catalytic rate of Pol δ

We have started to identify the essential amino acid residues on PCNA that may be involved in the stimulation of the intrinsic catalytic rate of Pol δ. We inspected the large sets of *POL30* (PCNA) mutants previously generated (23,24,43), with a focus on those mutants that show defects in cell growth and sensitivity to the replication inhibitor hydroxyurea. Many of those mutants also show defects in specific DNA repair pathways. We focused our study on pcna-6 (DD41,42AA). Pcna-6 shows a minor defect in cell growth, but a high sensitivity to hydroxyurea (24). The crystal structures of PCNA (44) and the pcna-6 mutant (45) are shown in Figure 4B. The main distinguishing characteristics are decreased hydrophilicity on the surface side that binds Pol δ (23,46–48). Previous biochemical studies showed that pcna-6 is loaded onto template/primer DNA by RFC, but its stimulation of DNA synthesis by Pol δ is compromised (24).

Initial replication studies on the model template/primer in Figure 4A indeed showed a defect in the rate of DNA replication with pcna-6 (data not shown). However, it was difficult to distinguish between a defect in the catalytic rate and a defect in processivity, or both. Therefore, we limited DNA replication by the PCNA•Pol δ complex to only a single processive cycle by inclusion of a heparin trap at *t* = 0, which inactivates Pol δ that dissociated from the DNA (9) (Figure 4A). Control experiments showed that heparin completely blocked Pol δ activity unless it was pre-bound to DNA (Supplementary Figure S4G).

We premixed template/primer DNA with Pol δ and with PCNA or pcna-6, along with RFC and AMP-CPP, and started the reaction by addition of dATP together with the heparin trap (Figure 4A). In the presence of wild-type PCNA, the final 49-mer product, resulting from the incorporation of 20 dAMPs was clearly apparent after 40–50 milliseconds (Figure 4C). However, the nucleotide extension reaction was much slower with mutant pcna-6, the 49-mer product only becoming visible after 70 milliseconds (Figure 4C). This replication rate defect is apparent at every time point. A comparison of the product distribution at 10 ms and 50 ms is shown in Figure 4D. At 10 ms, the peak of the normalized band intensities centers around four nucleotides for PCNA, but only around 1–2 nucleotides for pcna-6. At 50 milliseconds, the peak centers around 16 nucleotides for PCNA and around seven nucleotides for pcna-6. In essence, while pcna-6 still stimulates the catalytic rate of Pol δ by several fold, the rate of the pcna-6•Pol δ complex is about half that of PCNA•Pol δ.

One possible complication with the multiple-nucleotide extension assay in Figure 4 is that the overall rate of replication is a composite of the individual rates of nucleotide incorporation and the rate of translocation. Translocation is the movement of the enzyme from the DNA_*n*_ to the DNA_*n*+1_ position, from the *n*+1 to the *n*+2 positon, and so on, after each successive chemical step. To eliminate complications in interpretation because of considerations of translocation, we returned to the single-nucleotide incorporation assay. However, in order to obtain an accurate measurement of the extension rate by the wild-type PCNA–DNA–Pol δ complex, we needed to lower the temperature of the assay to 12°C (Figure 5). At this temperature, the rate with wild type PCNA was 183 s^−1^. Remarkably, the rate with the pcna-6 complex was only a third that of wild-type, at 55 s^−1^. In comparison, the rate without PCNA was measured at 6 s^−1^. Thus, at 12°C, wild type PCNA accelerates the rate of Pol δ by 30-fold while that by mutant pcna-6 is reduced to 9-fold.

### Considerations of Pol δ replication rates

The total of our data are consistent with a model in which PCNA stimulates a step in catalysis by the ternary DNA-Pol δ-dNTP complex. The rate constants k1 and k2 in our minimal kinetic scheme can be eliminated from consideration because the DNA was pre-bound with polymerase prior to addition of dNTP, and secondly, saturating concentrations of dNTPs were used. Therefore, PCNA either affects the conformational change of the ternary DNA•Pol•dNTP complex from the open to closed conformation (k3), or the chemical step (k4). We propose that the PCNA accelerates the rate of conformational change, and pcna-6 is partially defective for this stimulation. An alternative possibility, that PCNA stimulates the actual chemical step (k4) is unlikely, because in the closed conformation, the polymerase active site is inaccessible (6). Furthermore, recent studies with Pol ϵ indicate that the catalytic step is not rate-limiting for that enzyme (42). Nevertheless, an indirect stimulation of the chemical step through long-range conformational changes cannot be excluded. Interestingly, a polymerization rate defect by PCNA•Pol δ was also observed when the [4Fe–4S] sulfur cluster that is resident in the C-terminal domain of the catalytic subunit (29), was oxidized from the +2 to the +3 form (49). Unfortunately, the nature of the experimental setup in that study did not allow us to determine whether this rate defect is in the stimulation by PCNA or whether the oxidized form of Pol δ alone shows a reduced catalytic rate. Currently, it is not clear whether Pol δ from other organisms also shows the same behaviour since pre-steady state kinetics have not been carried out with those enzymes.

Human Pol δ is a 4-subunit enzyme: p125^Pol3^-p50^Pol31^-p68^Pol32^-p12, in which the fourth (p12) subunit is regulated during DNA damage (50). The analogous *S. pombe* fourth subunit (*Cdm1^+^*) is dispensable for fission yeast cell growth, displaying no detectable replication or repair phenotypes (51). Different 3-subunit assemblies of human Pol δ have been reconstituted (52). Interestingly, in the presence of PCNA, the catalytic rates of the 4-subunit assembly and the 3-subunit p125^Pol3^-p50^Pol31^-p12 assembly are 3-fold higher than that of the 3-subunit p125^Pol3^-p50^Pol31^-p68^Pol32^ assembly. Whether these differences are processivity-related or whether p12 actually affects the catalytic rate of the polymerase remains to be determined.

## Supplementary Material

Supplementary Data
