# Supplementary material for: PCNA accelerates the nucleotide incorporation rate by DNA polymerase δ
Source: Nucleic Acids Res. 2019 Jan 3;47(4):1977–86. doi: 10.1093/nar/gky1321 (PMC6393303; doi:10.1093/nar/gky1321)
Supplement: Supplementary Data [file gky1321_supplemental_files.docx]

**SUPPLEMENTARY DATA**

**PCNA accelerates the nucleotide incorporation rate by DNA polymerase δ**

**Tanumoy Mondol^1^, Joseph L Stodola^1,2^, Roberto Galletto^1^ and Peter M Burgers^1, *^**


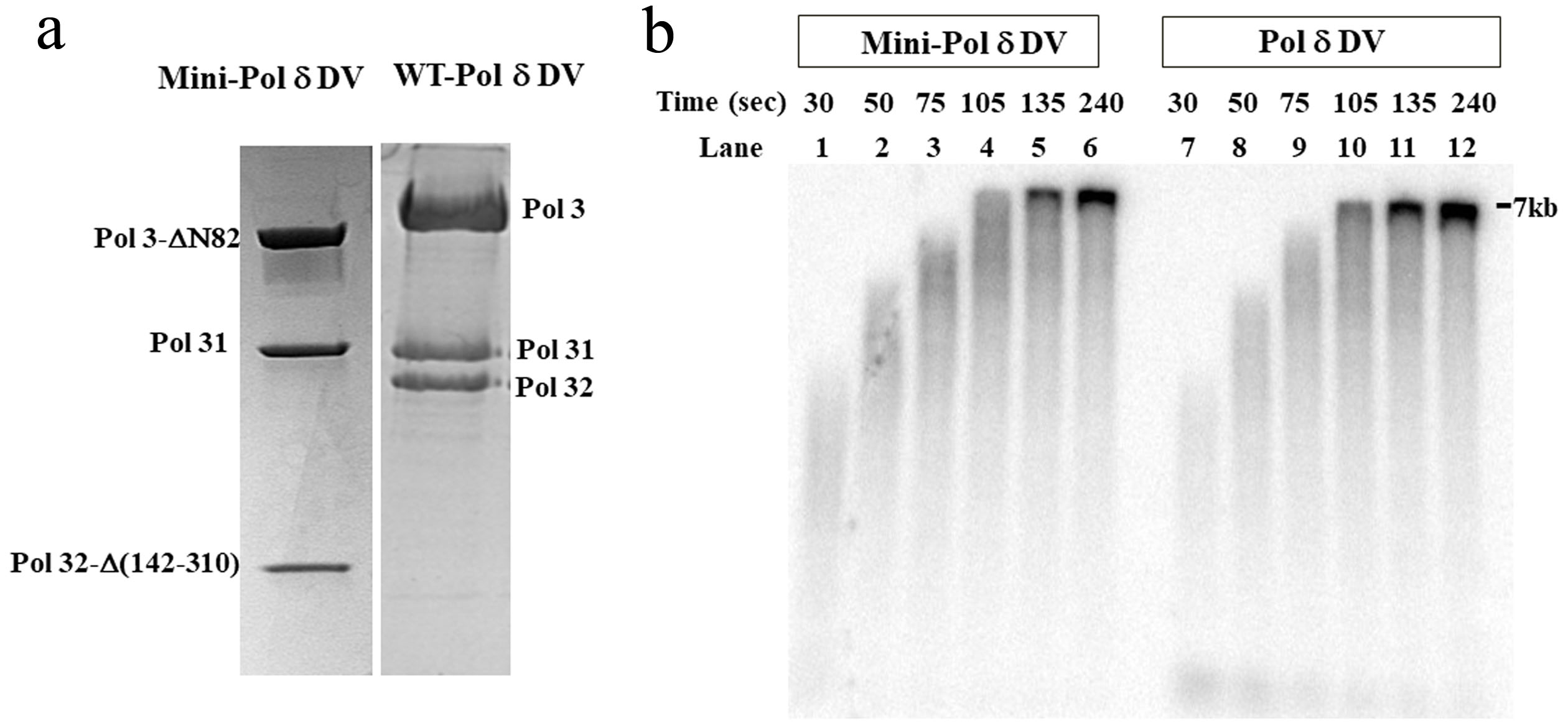


**Supplementary Figure 1:** (a) Coomassie-stained SDS-PAGE of mini-Pol δ-DV and Pol δ-DV, purified from yeast. (b) Comparison of the replication efficiency of mini-Pol-δ DV and Pol-δ DV. Pol δ-DV and mini-Pol δ-DV holoenzyme assay on M13mp18 DNA. Assays contained 40 mM Tris-HCl (pH 7.8), 1 mM dithiothreitol (DTT), 100 μg/ml bovine serum albumin, 8 mM magnesium acetate, 0.5 mM ATP, 100 μM each of dCTP, dGTP and dTTP, 10 μM of [α−^32^P]dATP, 100 mM NaCl, 2 nM singly-primed single-stranded M13mp18 DNA, 500 nM of RPA and 5 nM PCNA. PCNA was loaded onto the primed DNA by incubation with 5 nM RFC at 30 ^0^C for 1 minute, and replication was started by adding mini-Pol δ-DV or Pol δ-DV. Aliquots were taken at various time points, and replication was stopped by the addition of 20 mM EDTA and 0.2% SDS final concentrations. After incubating at 50^0^C for 10 minutes, the products were electrophoresed on a 0.8% alkaline agarose gel at 30 V for 14-15 hours. Gels were neutralized, dried and exposed to a phosphor screen, and visualized on a Typhoon phosphorimager (GE Healthcare). The migration position of the complete replication product is indicated.

**
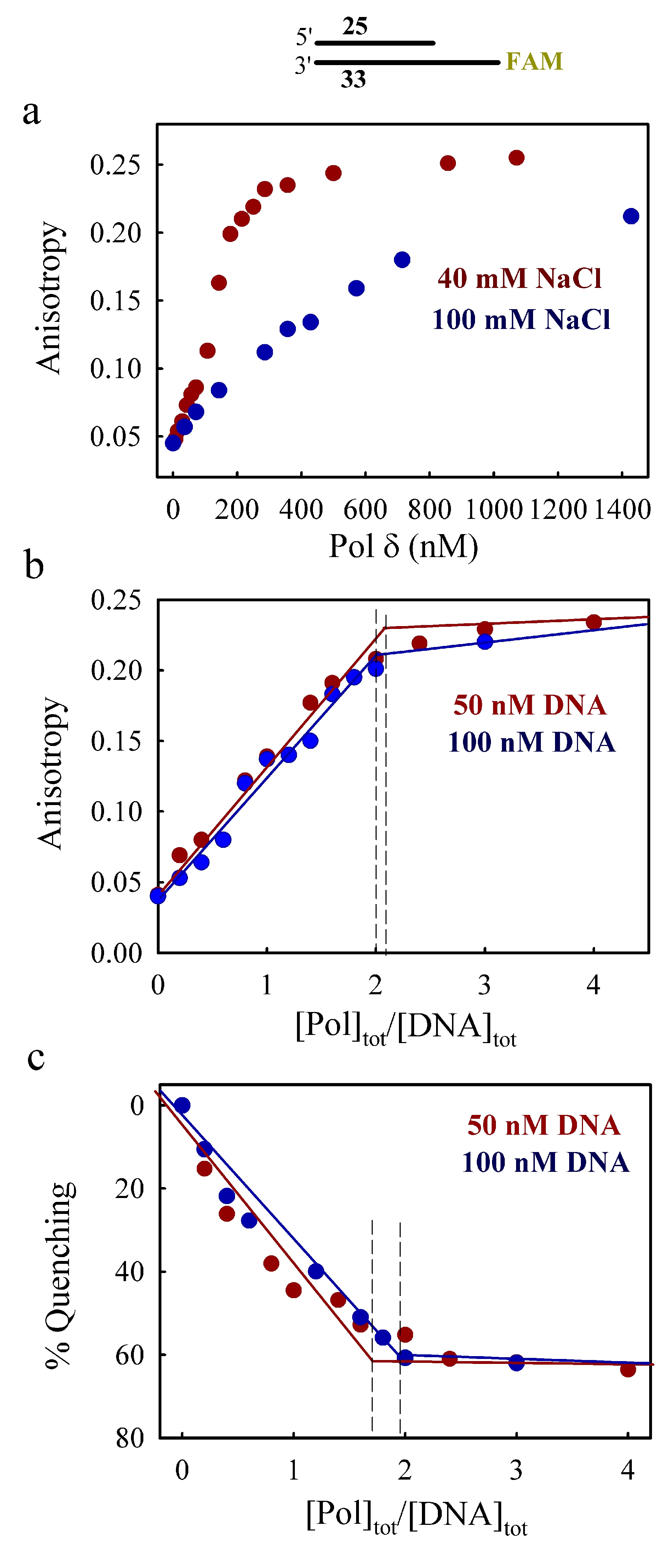
**

**Supplementary Figure 2**. DNA binding experiments with 33/25 template/primer shown on top. (a) Increase in fluorescence anisotropy of 100 nM DNA with increasing concentrations of mini-Pol δ-DV at 100 mM NaCl (blue) or 40 mM NaCl (red). (b) Fluorescence anisotropy of either 50 nM (red) or 100 nM (blue) DNA with increasing concentrations of full-size Pol δ-DV at 40 mM NaCl. (c) As in (b) for fluorescence quenching. The data points are fitted to a two-segment fit analysis (using Origin software). Vertical dotted lines were drawn from the intersection points.

**
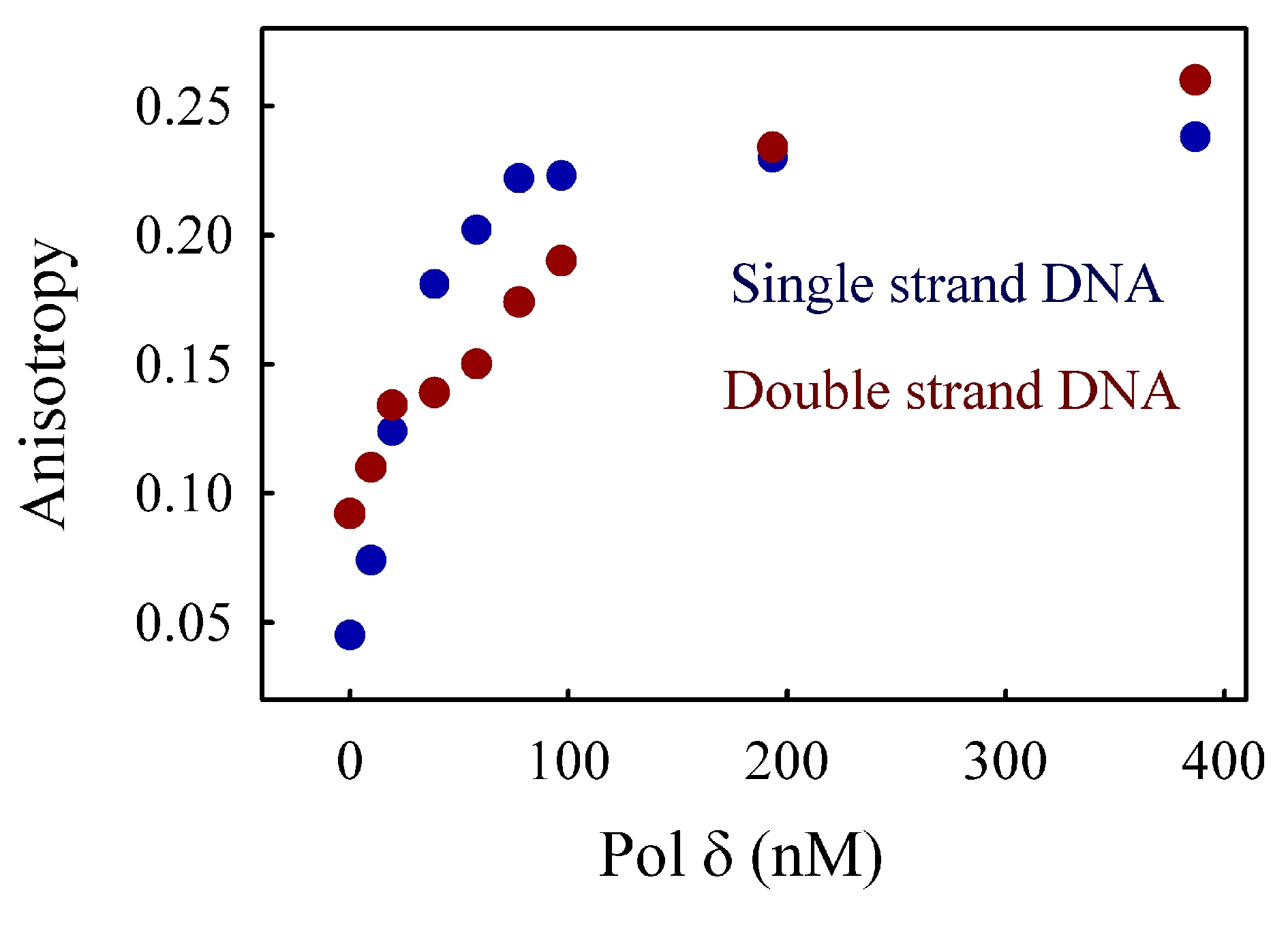
**

**Supplementary Figure 3**: Fluorescence anisotropy binding titration of mini-Pol δ-DV to single stranded and double stranded DNA. Increase in anisotropy of 50 nM single strandeded DNA (33 mer; blue) or 50 nM double stranded DNA (33 mer; red) with increasing mini-Pol δ-DV concentration at 40 mM NaCl.


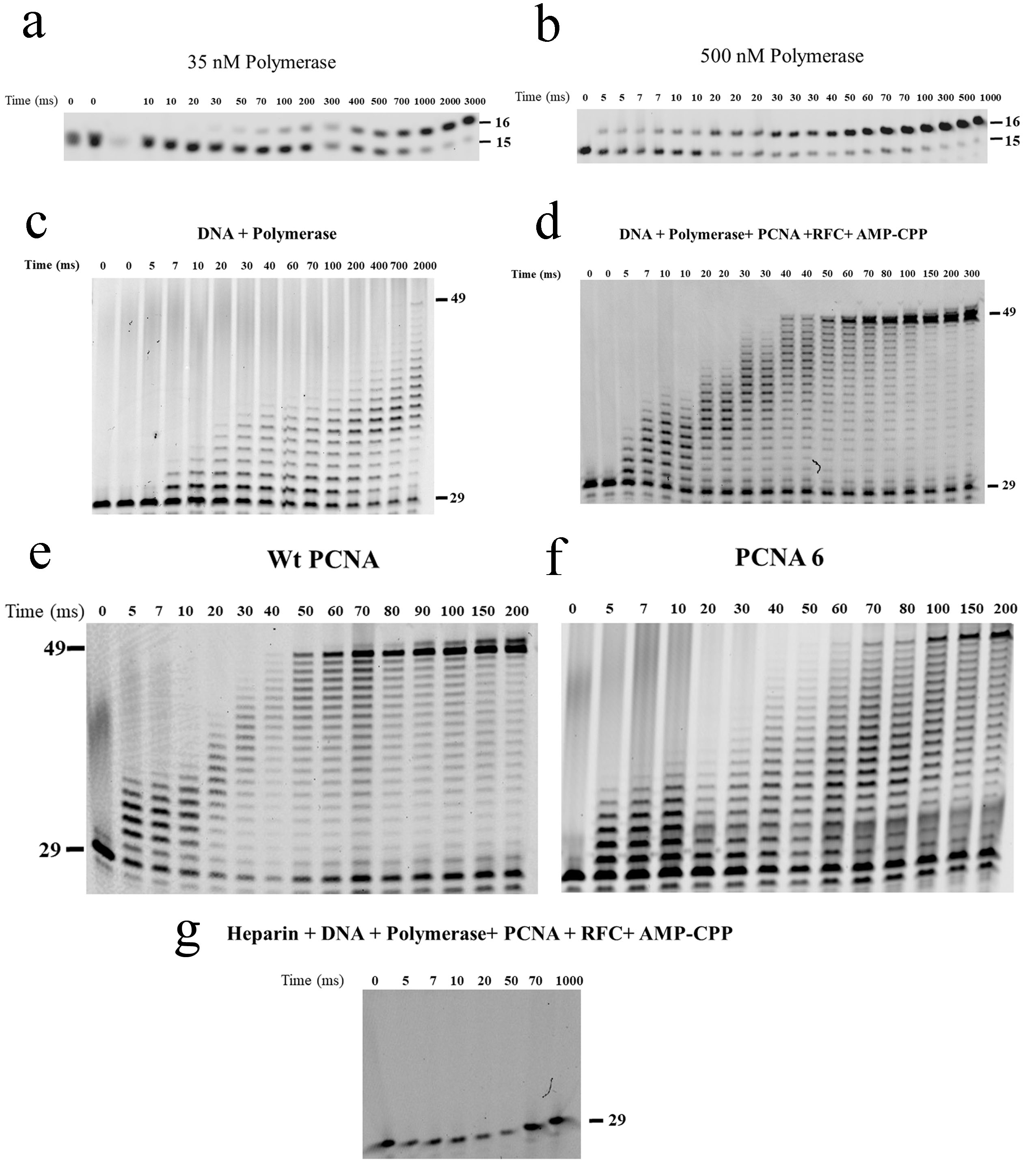


**Supplementary Figure 4**: Time courses of single nucleotide incorporation by (a) 35 nM and (b) 500 nM mini-Pol δ-DV pre-incubated with 50 nM template primer DNA (23/15 mer). Reaction was initiated with 250 μM final dTTP allowing extension of 15 mer primer to 16 mer product. The 10, 20, 30, 50, 70, 100, 300, 500, 1000, and 3000 msec lanes of a) were used in Figure 2b, and the 7, 10, 20, 30, 50, 70, 100, 300, 500, and 1000 msec lanes of b) were used in Figure 2b. (c, d) Time courses of primer extension by 300 nM mini-Pol δ-DV pre-incubated with 50 nM template primer DNA (75/29 mer), (c) in the absence of PCNA, RFC and AMP-CPP and (d) in presence of 150 nM PCNA, 75 nM RFC and 100 μM AMP-CPP. Both reactions were initiated with 250 μM final dATP. (e, f) Time courses of primer extension by mini-Pol δ-DV (300 nM) pre-incubated with 50 nM template primer DNA (75/29 mer) in the presence of 75 nM RFC and 100 μM AMP-CPP, and (e) 150 nM PCNA, or (f) 150 nM pcna-6. Both reactions were initiated with 250 μM final dATP and 17 μg/ml final heparin. (g) Control assay showing the efficacy of the heparin trap. Mini-Pol δ-DV was preincubated with DNA, PCNA, RFC, and AMP-CPP as in c-f, together with heparin, and then mixed with an equal volume of dATP to intiate synthesis. None was observed.

**SUPPLEMENTARY TABLE 1**

Nucleotide incorporation rates (*K*_pol_) obtained at different polymerase concentration. The data for 500 nM enzyme were fitted to a single exponential function: 1-exp(-k_fast_*t); the data for 35-150 nM enzyme were fitted to a double exponential function: A(1-exp(-40*t)+ (1-A)(1-exp(-k_slow_*t). the curves were plotted in Figure 2c.

| Polymerase (nM) | *k*_fast_ (sec^-1^) | *k*_slow_ (sec^-1^) |
| --- | --- | --- |
| 35 | 40 (8%) | 1.5 (92%) |
| 75 | 40 (47%) | 4.3 (53%) |
| 150 | 40 (81%) | 6.0 (19%) |
| 500 | 40 (100%) | - |
